# Energy expenditure during common sitting and standing tasks: examining the 1.5 MET definition of sedentary behaviour

**DOI:** 10.1186/s12889-015-1851-x

**Published:** 2015-05-29

**Authors:** Maedeh Mansoubi, Natalie Pearson, Stacy A Clemes, Stuart JH Biddle, Danielle H Bodicoat, Keith Tolfrey, Charlotte L Edwardson, Thomas Yates

**Affiliations:** School of Sport, Exercise & Health Sciences, Loughborough University, Loughborough, Leicestershire, LE11 3TU, UK; The NIHR Leicester-Loughborough Diet, Lifestyle and Physical Activity Biomedical Research Unit, Leicester-Loughborough, Leicestershire UK; Institute of Sport, Exercise & Active Living, Victoria University, Melbourne, Australia; Leicester Diabetes Centre, Leicester General Hospital, University of Leicester, Leicester, Leicestershire, UK

**Keywords:** MET, Energy expenditure, Sedentary behavior, Physical activity

## Abstract

**Background:**

Sedentary behavior is defined as any waking behavior characterized by an energy expenditure of 1.5 METS or less while in a sitting or reclining posture. This study examines this definition by assessing the energy cost (METs) of common sitting, standing and walking tasks.

**Methods:**

Fifty one adults spent 10 min during each activity in a variety of sitting tasks (watching TV, Playing on the Wii, Playing on the PlayStation Portable (PSP) and typing) and non-sedentary tasks (standing still, walking at 0.2, 0.4, 0.6, 0.8, 1.0, 1.2, 1.4, and 1.6 mph). Activities were completed on the same day in a random order following an assessment of resting metabolic rate (RMR). A portable gas analyzer was used to measure oxygen uptake, and data were converted to units of energy expenditure (METs).

**Results:**

Average of standardized MET values for screen-based sitting tasks were: 1.33 (SD: 0.24) METS (TV), 1.41 (SD: 0.28) (PSP), and 1.45 (SD: 0.32) (Typing). The more active, yet still seated, games on the Wii yielded an average of 2.06 (SD: 0.5) METS. Standing still yielded an average of 1.59 (SD: 0.37) METs. Walking MET values increased incrementally with speed from 2.17 to 2.99 (SD: 0.5 - 0.69) METs.

**Conclusions:**

The suggested 1.5 MET threshold for sedentary behaviors seems reasonable however some sitting based activities may be classified as non-sedentary. The effect of this on the definition of sedentary behavior and associations with metabolic health needs further investigation.

## Background

Over the past few decades, the way in which we live our everyday lives has changed dramatically. Technological advances, societal influences and environmental attributes have significantly influenced the way we socialize, travel, work and shop resulting in substantial proportions of the day spent in sedentary pursuits, or sitting [1]. A growing body of epidemiological evidence has linked sedentary behavior to health risks including an increased risk of type 2 diabetes [2, 3], the metabolic syndrome [4], cancer [5, 6], obesity [7, 8] and all-cause and CVD mortality [3, 6, 9]. These associations have been shown to be at least partially independent of physical activity, suggesting that sedentary behaviors have the potential to influence risk of disease, independent of physical activity levels. Furthermore, recent reviews have noted that there is an inverse association between some sedentary behaviors (mostly TV viewing or screen time) and leisure-time physical activity in adults [10, 11], providing evidence for time displacement.

Such evidence requires us to examine sedentary behavior as a concept in itself and there are a growing number of analytical considerations regarding what constitutes sedentary behavior [12]. Sedentary behavior is not simply a lack of physical activity or a failure to meet recommended levels of moderate-to-vigorous physical activity [13–16], this should be defined as ‘inactivity’ [12]. Sedentary behavior has recently been defined as “*any waking behavior characterized by an energy expenditure of ≤1.5 METs while in a sitting or reclining posture*” (page 540) [12]. This definition acknowledges the importance of posture but also energy expenditure in defining sedentary activities. However, the utility of the 1.5 MET threshold is poorly understood. For example, in the compendium of physical activities [17], sedentary activities, such as; sitting at a desk, sitting in a vehicle, sitting watching television, have been coded with MET values ranging from 1.0-2.5, but standing activities, such as watering the lawn or garden, which are not classified as sedentary in the above definition due to the upright posture in which they are performed, are coded with a MET value of 1.5 [17]. In addition, playing computer games (often categorized as sedentary behaviors in self-report questionnaires) have been found to have MET values as high as 4.5 [18].

Limited studies [19, 20] have examined the differences in energy cost of lifestyle activities in healthy weight, overweight and obese adults. It has been shown that the energy cost of activities such as walking could be predicted by body weight [19, 20]. However the examination of the MET definition of sedentary behavior is required across body composition groups to ascertain the widespread applicability of this definition. The aims of this study were therefore (a) to measure energy expenditure during common sitting, standing and walking tasks and (b) to examine the utility of the 1.5 MET definition of sedentary behavior in distinguishing between common sitting and standing activities in healthy weight and obese participants.

## Methods

### Study design

**Fig. 1:**
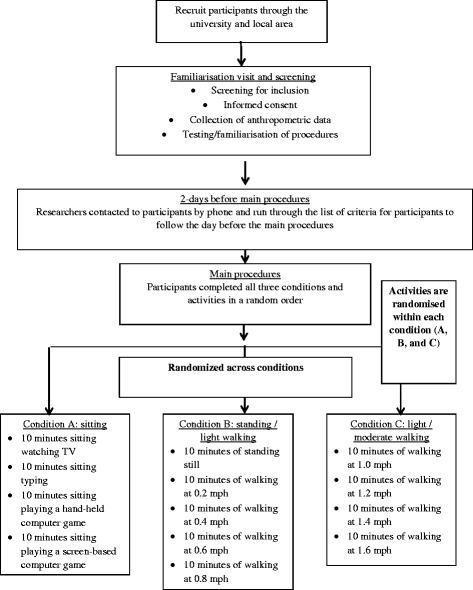
Flowchart for the participants included in the study

This study was an experimental cross-over trial (Fig. 1 shows flow of participants through the study). In total, there were three conditions (sitting, standing/very light walking, and light-moderate walking). Within each condition there were several activities (see Fig. 1). All participants completed each of the conditions and each of the associated activities. The order of the conditions and activities were randomized. First, participants were randomized to the order in which they undertook each of the three conditions with stratification for BMI (healthy weight vs. obese) and sex (male vs. female). Second, participants were randomized to the order in which they undertook each of the activities within the three conditions, without any further stratification. The study was approved by the Loughborough University Ethical Advisory committee and all participants provided written informed consent.

### Participants and recruitment

Recruitment was purposefully undertaken based on a 2 × 2 format that required equal numbers of male/female and healthy weight (BMI < 25 kg/m^2^) and obese (BMI > 30 kg/m^2^) participants, over the age of 18 years. Recruitment was undertaken within Loughborough University and the local community through email and posters. People who displayed an interest in participating in the study received a study information pack detailing the study and requirements. Exclusion criteria included the presence of any physical conditions or illnesses which prevented full participation in the study, or an inability to communicate in spoken English.

### Sample size

To detect a significant association between walking speed and MET level, in order to evaluate how MET levels increase from standing to light walking activities, a minimum of 9 participants per group (male, female) was required. This was based on assuming 1-beta = 0.8, alpha = 0.05 and R^2^ = 0.6 [18].

### Familiarization visit and screening

Potential participants were invited to the laboratory at least 10-days before the main test for a familiarization visit. During this visit, participants were screened for inclusion/exclusion criteria into the study. Eligible participants were shown the designated experimental area and provided with an opportunity to try some of the experimental activities (e.g. walking on the treadmill), familiarize themselves with the gas mask, and ask questions about the protocol. During this visit, anthropometric measures were taken which included height (measured using a portable stadiometer, Seca UK), waist circumference (measured mid-way between the lower rib margin and the iliac crest using anthropometry tape) and body weight and composition (measured using a Tanita Body Composition Analyzer, model: BC-418 MA, Tanita, UK).

### Experimental protocol

As energy expenditure increases substantially during and following vigorous physical activity, and as recovery time varies depending on the intensity, duration, type of activity, and fitness level of the individual, participants were asked to refrain from vigorous activity for 48 h before attending the laboratory [21]. In addition, due to their influences on resting metabolic rate, participants were also asked to abstain from caffeine and alcohol for 36 h before the experimental protocol [21]. Participants were asked to consume their usual evening meal between 17:00 and 19:30, and they were given a snack (cereal bar) to eat up until 20:00 the day before attending the laboratory. Participants arrived at the laboratory at 08:00 following an overnight fast, with only water consumed from 20:00 the evening before. Participants were asked to arrive by car to eliminate uncontrolled activity and 500 mL of water was consumed at least 60 min before arriving at the laboratory.

Upon confirmation that participants had complied with the pre-study requirements, participants’ spent 60 min in a semi-supine resting position under a ventilated hood (GE Nutrition. UK). Resting Metabolic Rate (RMR) was quantified over the second 30 min period. Following the RMR measures under the ventilated hood, participants wore a face-mask, which was connected to an open circuit breath-by-breath automated gas-analysis system measuring expired respiratory gas fractions (Cortex Metalyzer, Leipzig, Germany). Participants spent a further 30 min in a semi-supine state to repeat the RMR measure with the breath-by-breath automated gas-analysis system. This was done to allow a comparison between measurement types (ventilated hood vs. breath-by-breath analyzer) to ensure consistency with measurements taken during the protocols described below.

Following the assessment of RMR participants consumed a standardized breakfast following recommendations from the Food Standards Agency (FSA) that breakfast should constitute 20 % of daily energy intake [22]. For males this consisted of: 80 g Weetabix minis cereal (299 kcal) + 250 mL semi-skimmed milk (123 kcal) + one banana (~80 kcal). For females the meal consisted of: 60 g Weetabix minis cereal (224 kcal) + 200 mL semi-skimmed milk (98 kcal) + one banana (~80 kcal). Following a 20 min rest period, participants performed a series of activities under three conditions (A - sitting, B - standing and very light walking, C - light walking).

The ‘sitting’ condition (condition A) involved the following four activities. 1) sitting watching television (TV: an episode of a TV drama shown to each participant), 2) sitting typing (each participant copied the same text from the same book), 3) sitting playing a hand-held computer game (participants played a tennis game with a PSP [PlayStation Portable, Sony]) and 4) sitting playing a TV screen-based computer game (participants played a tennis game with the Wii). The ‘standing and very light walking’ condition (condition B) included five activities: standing still (participants asked to stand as if they were waiting in a queue/line) and light walking at 0.2, 0.4, 0.6, and 0.8 miles/h on a treadmill (Technogym, Excite Med, UK). The ‘light-moderate walking’ condition (condition C) involved participants walking on a treadmill at 1.0, 1.2, 1.4, and 1.6 miles/h. Each activity within the three conditions was conducted for 10 min, with expired gas collected during the last 5 min. In between each condition (A, B and C), participants were offered a 5-min break to remove the face-mask for their comfort. Respiratory gas was collected using the Cortex breath-by-breath automated gas-analysis system.

### Statistical analyses

To achieve the primary aim, summary measures of the MET values associated with each activity were produced. The MET values were calculated using the standardized MET formula: MET = VO_2_ (mL/kg/min)/3.5 (mL/kg/min). We also derived a second index by calculating multiples of resting metabolic rate (mRMR) by dividing VO_2_ during each activity by VO_2_ at rest; unlike standardized METs, mRMR takes into account individual differences in VO_2_ during rest. The Shapiro-Wilk test confirmed that all data were normally distributed. Differences between the BMI groups were tested using independent t-tests. Generalized Estimating Equations (GEE) were used to determine the association between walking speed and average MET values. These models took into account the repeated measurements taken on the same individuals [23]. P < 0.05 was considered significant and all tests were 2-sided. All statistical analyses were performed using SPSS version 22 (IBM SPSS Statistics). Data are displayed as mean (± SD) and mean (95 % CI) in the text and tables.

## Results

Fifty-one adults (25 males [13 healthy weight and 12 obese) and 26 females [14 healthy weight and 12 obese]) completed the laboratory protocol. The characteristics of the participants are displayed in Table 1.

**Table 1:** Descriptive anthropometry data (mean [SD]) for the healthy weight and obese groups, stratified by sex

Group	Sex	Body mass (kg)	Stature (m)	BMI (kg/m^2^)	Body fat (%)	Waist circumference (cm)	Age (years)
Healthy Weight	Male (n = 13)	70.4 (6.5)	174.8 (7.3)	23.1 (1.5)	16.5 (3.5)	84.8 (1.5)	32.7 (13.8)
	Female (n = 14)	57.8 (7.3)	161.9 (6.5)	22.01 (1.9)	23.7 (6.7)	72.1 (1.9)	29.1 (3.6)
Obese	Male (n = 12)	92.9 (9.4)	170.7 (8.6)	31.8 (1.8)	29.1 (7.5)	104.4 (5.8)	38.2 (14.6)
	Female (n = 12)	91.6 (12.8)	164.4 (9.7)	33.8 (3.8)	38.4 (8.9)	104.3 (11.2)	32.5 (12)

### Resting metabolic rates

The mean (SD) absolute VO_2_ level measured by the GEM ventilated hood for the whole sample was 245 (44) mL/min. Resting values were slightly higher in the obese participants (256 (49) mL/min) in comparison to the healthy weight participants (235 (38) mL/min). After adjusting the results for participants’ body mass, mean (SD) VO_2_ for the whole sample was 3.28 (0.74) mL/kg/min (obese participants: 2.81 (0.62) mL/min/kg; healthy weight participants: 3.71 (0.58) mL/kg/min). Resting VO_2_ values were similar when using the Cortex calorimeter (3.28 (0.29) mL/kg/min), no significant differences between methods were observed (p = 0.959).

### mRMR and MET values of different activities

For the whole sample, mean (SD) standardized MET values for inactive sitting tasks ranged from 1.33 (0.24) to 1.45 (0.32), see Table 2. The more active, yet still seated, games on the Wii yielded an average of 2.06 (0.50) METS. Standing yielded an average of 1.59 (0.37) METs. Walking MET values increased incrementally with speed from 2.17 (0.5) at 0.2 miles/h to 3.22 (0.69) METs at 1.6 miles/h.

**Table 2:** Metabolic rate during sitting activities and slow walking for the sample as a whole, and for the healthy weight and obese groups separately. Standard MET values and the mRMR values are shown, along with the results of the independent t-tests comparing MET values between healthy weight and obese participants

	Standard MET values of sitting, standing and light walking tasks				mRMR values of sitting, standing and light walking tasks			
Activity	Total MET	Healthy Weight MET	Obese MET	P value for between group difference	Total MET	Healthy Weight MET	Obese MET	P Value for between group difference
TV	1.33 (0.24)	1.46 (0.19)	1.17 (0.20)	p < 0.001	1.45 (0.27)	1.40 (0.20)	1.51 (0.33)	0.151
Typing	1.45 (0.32)	1.62 (0.23)	1.23 (0.28)	p < 0.001	1.56 (0.16)	1.57 (0.18)	1.54 (0.16)	0.651
PSP	1.41 (0.28)	1.58 (0.21)	1.21 (0.22)	p < 0.001	1.52 (0.16)	1.50 (0.13)	1.54 (0.20)	0.358
Wii	2.06 (0.50)	2.29 (0.44)	1.80 (0.44)	p < 0.001	2.22 (0.43)	2.18 (0.41)	2.28 (0.46)	0.401
Standing Still	1.59 (0.37)	1.74 (0.34)	1.41 (0.33)	p < 0.001	1.71 (0.29)	1.65 (0.22)	1.78 (0.33)	0.105
Speed (mph)	Treadmill Walking							
0.2	2.17 (0.5)	2.44 (0.44)	1.87 (0.40)	p < 0.001	2.33 (0.28)	2.31 (0.28)	2.36 (0.29)	0.509
0.4	2.27 (0.26)	2.56 (0.49)	1.94 (0.44)	p < 0.001	2.43 (0.28)	2.41 (0.25)	2.44 (0.33)	0.788
0.6	2.40 (0.54)	2.67 (0.46)	2.09 (0.)	p < 0.001	2.58 (0.32)	2.52 (0.26)	2.63 (0.38)	0.228
0.8	2.55 (0.62)	2.84 (0.54)	2.21 (0.53)	p < 0.001	2.73 (0.37)	2.69 (0.34)	2.77 (0.41)	0.411
1.0	2.66 (0.61)	2.97 (0.46)	2.32 (0.57)	p < 0.001	2.85 (0.34)	2.81 (0.33)	2.89 (0.35)	0.401
1.2	2.83 (0.67)	3.17 (0.55)	2.45 (0.59)	p < 0.001	3.03 (0.36)	2.99 (0.32)	3.06 (0.41)	0.543
1.4	2.99 (0.69)	3.30 (0.57)	2.63 (0.64)	p < 0.001	3.21 (0.39)	3.12 (0.35)	3.30 (0.42)	0.150
1.6	3.22 (0.53)	3.60 (0.70)	2.80 (0.63)	p < 0.001	3.46 (0.54)	3.41 (0.55)	3.53 (0.52)	0.439

Mean (SD) mRMR values for inactive sitting tasks ranged from 1.45 to 1.56 (0.27–0.65) METs. Active seated games on the Wii yielded an average of 2.2 (0.43) METS (see Table 2). Standing yielded an average of 1.71 (0.29) METs. Walking MET values increased incrementally with speed from 2.33 (0.28) to 3.46 (0.54) METs.

mRMR values were not significantly different between healthy weight and obese participants nor between males and females for any activities (Table 2). However, for standardized METS in all activities there were significant differences between obese and healthy weight participants (Table 2). Obese participants had significantly lower MET values for all activities. There was no significant differences between male and female MET values (p > 0.05) (data not shown). Generalized Estimating Equations (GEE) showed that walking speed predicted standardized MET values. Each 1 mile/h increase in walking speed was associated with a 0.79 (p < 0.001) increase in MET value. These values were not modified by obesity status. (Table 3).

**Table 3:** Results of the Generalized Estimating Equations (GEE) showing the associations between obesity and walking speed and predicted standardized MET values

		95% Confidence interval		P value
Parameter	B	Lower	Upper	Sig.
Obesity	0.152	−0.026	0.330	0.095
Speed	0.792	0.751	0.832	p < 0.001

## Discussion

Sedentary behavior has been defined as any waking behavior characterized by an energy expenditure of ≤1.5 METs while in a sitting or reclining posture [12]. Our study broadly supports this definition, but suggests that some common sitting behaviors, such as playing a Wii computer game or typing in normal weight individuals, may have a MET value above this threshold and are thus technically defined as non-sedentary activities in such instances. Conversely, standing behaviors may actually have MET values below 1.5 when accompanied by no ambulation particularly in obese participants. MET values also increased rapidly with walking speed so that every increase in walking speed of 1 mph increased MET values by 0.79. Standard MET values were significantly different between obese and healthy weight individuals during all conditions, but not between males and females. When standardized against resting metabolic rate, there were no significant differences in MET values (mRMR) between the healthy weight and obese groups, or between males and females.

Our results are broadly consistent with other studies which have measured sitting energy expenditure. These studies have shown that inactive sitting based activities (such as TV viewing) have MET values below 1.5 [24–28]. Our finding that metabolic activity during standing is similar or even lower than some sitting activities is consistent with other studies which have shown no significant differences between sitting and standing MET values [29, 30]. Taken together, these results have important implications. Sedentary behaviors have been strongly linked to metabolic health, morbidity and mortality with experimental research confirming the benefit of breaking sedentary behavior with bouts of light walking [31]. But it is currently unknown whether the positive benefits of reduced sedentary behavior are primarily driven by increases in energy expenditure that accompany the transition into light activity, or to differences in postural allocation, or a combination of both [32].

These results suggest that the energy gap between many sitting activities and standing without ambulation may be negligible; therefore suggesting differences in energy expenditure may be unlikely to explain any metabolic advantages of substituting sitting for standing based activities, unless accompanied by light movement or ambulation. We add to existing data by showing that any form of ambulation substantially elevates VO_2_ and accompanying MET values even at very low speeds of walking such as 0.2 mph. This finding could be very important for behavioral change interventions which promote standing with very light movement. Such interventions may be feasible in the workplace to reduce, and break up, prolonged sitting in those with predominantly sedentary occupations, such as office workers [1, 32].

The present results show that there were significant differences in standardized MET values between healthy weight and obese participants during all activities. This study therefore emphasizes the limitation of using a standardized resting VO_2_ value of 3.5 ml/kg/min across all individuals. Although obese individuals have a higher absolute VO_2_ value, the values per kg of body weight tends to be substantially lower than healthy weight individuals given that adipose tissue is less metabolically active than lean body mass. These findings are consistent with other studies that highlight the limitations of using a standardized number of 3.5 (mL/kg/min) for calculating metabolic rate [19, 20]. These findings could have important implications when METs are used for evaluating or prescribing physical activity intensity category (light, < 3 METs; moderate, 3–6 METs; vigorous, > 6 METs) [33]. Using a standardized equation which is not adjusted for personal differences, could also affect the MET compendium [17]. For example in our study with the mRMR equation, moderate activity begins after a speed of 1.2 mph but with the standardized equation, moderate physical activity was observed at a walking speed of 1.6 mph. However, it should be noted that mRMR values were consistent across healthy weight and obese individuals which in turn were similar to the standardized MET values for healthy weight individuals. Therefore, although of academic interest, these finding do not justify the need to reclassify activity METs or intensity thresholds for different groups and suggest that standardized values give a good indication of the degree to which RMR are elevated across different body weight ranges.

This study showed that with every 1 mph increase in walking speed, metabolic rate will significantly increase by 0.79 METs, and this was the same for healthy weight and obese participants. This result is consistent with other studies which published regression equations to predict walking (Km/h) METs and energy expenditures [34, 35], across different body size groups [36, 20].

It was observed in this study that no significant differences in RMR were observed between the GEM ventilated hood and the Cortex calorimeter. To our knowledge, this is the first study to compare the Cortex calorimeter against a gold standard ventilated hood. These findings confirm the validity of the measures of energy expenditure taken by the Cortex in the main laboratory protocol. Findings also support the use of the Cortex calorimeter in studies where the assessments of RMR using a ventilated hood are not feasible.

The limitations of this study include having a relatively small sample and the assessment of a limited number of activities. Study strengths however include the novel comparison of energy expenditures during some lifestyle activities in a stratified sample of males and females, and healthy weight and obese adults.

## Conclusion

The 1.5 MET threshold seems to be reasonable at distinguishing between most sitting and standing behaviors, however some common sitting behaviors appeared to have a MET level above this threshold. These findings have specific relevance to the current definition of sedentary behavior and suggest that common sitting activities, such as typing, are actually defined as non-sedentary in a large proportion of the population. The implications of this need exploring further. Research is also needed to unpick the minimum amount of ambulatory activity that needs to accompany standing in order to provide clinically meaningful benefits.
